# Immediate Breast Reconstruction of a Nipple Areolar Lumpectomy Defect With the L-Flap Skin Paddle Breast Reduction Design and Contralateral Reduction Mammoplasty Symmetry Procedure: Optimizing the Oncoplastic Surgery Multispecialty Approach

**Published:** 2017-03-31

**Authors:** Mitchell Buller, Adee Heiman, Jared Davis, Thomas J. Lee, Nicolás Ajkay, Bradon J. Wilhelmi

**Affiliations:** ^a^School of Medicine, University of Louisville; ^b^Division of Plastic and Reconstructive Surgery; ^c^Division of Surgical Oncology, Department of Surgery, University of Louisville, Louisville, Ky

**Keywords:** oncoplastic, mammoplasty, areola, lumpectomy, symmetry

## Abstract

**Objective:** We describe a modification of the inferior pedicle reduction mammoplasty for oncoplastic reconstruction of a central tumor defect. Our technique involved a deepithelialized L-shaped medial inferior based flap with removal of lateral breast tissue after central lumpectomy with a contralateral Wise-pattern mastopexy with inferior pedicle for symmetry. This technique is ideal for patients with large, ptotic breasts that desire breast conservation with immediate reconstruction. **Methods:** A 47-year-old woman with size 38 DD breasts presented with a palpable 2-cm subareolar mass of the left breast. Surgical oncology performed a left lumpectomy with nipple-areola complex excision and a sentinel lymph node biopsy. Immediate left breast reconstruction was performed with an inferior pedicle island flap. An additional 30 g of breast tissue was excised laterally for contour, and the neo–nipple-areola complex was rotated into the defect to facilitate inverted-T closure. A standard Wise-pattern mastopexy with inferior pedicle was then performed on the right breast and an additional 205 g of tissue was removed for symmetry. **Results:** The patient showed excellent symmetry at the conclusion of the procedure. Final pathology demonstrated complete excision of the tumor with negative margins. The entire neo–nipple-areola complex skin island was viable postoperatively. **Conclusions:** Immediate reconstruction of a nipple-areola complex lumpectomy defect with a L-shaped medial inferior based skin paddle flap and contralateral reduction mammoplasty provides an excellent cosmetic outcome in patients with large, ptotic breasts and central defects following oncologic tumor resection.

Central or retroareolar breast cancer accounts for 5% to 20% of all cases of breast cancer.^1^ Previous studies have shown that central tumors within 2 cm of the nipple have an increased rate of occult nipple malignancy and microscopic infiltration has been present in up to 50% of cases.^2^^,^^3^ For this reason, tumor proximity to the nipple-areola complex (NAC) has been considered an indication for NAC removal. As the NAC is a defining feature of the breast, these patients were traditionally not considered for breast conservation.^4^ Oncoplastic techniques have since been described to provide more favorable reconstructive results.

Simple techniques for closure of a central defect involving the NAC include direct closure of a round or spindle-shaped defect, and a wise-pattern, inverted-T closure.^5^ Many techniques for nipple reconstruction have been well described, such as purse string closure local flaps, or tattooing.^5^^,^^6^ There are also more sophisticated techniques for immediate breast mound and NAC reconstruction. These can be classified as volume displacement or replacement techniques.

Volume replacement techniques, such as the latissimus dorsi myocutaneous flap and the AICAP flap, are preferable in patients with smaller, nonptotic breasts,^1^^,^^7^^-^9 whereas patients with large ptotic breast are favorable for volume displacement techniques.^1^ Volume displacement is based on principles of mastopexy and therefore it is easily reproducible on the contralateral side.^5^ Such techniques include the inferior pedicle reduction mammoplasty,^5^^,^^7^ the Grisotti flap,^1^^,^^2^^,^^8^^,^^10^ and the Hall-Findlay technique.^11^ All these techniques involve a deepithelialized flap, with creation of a circular skin island to replace the NAC defect.

We describe a modification of the inferior pedicle reduction mammoplasty. Our technique involved a deepithelialized L-shaped flap with removal of lateral breast tissue after central lumpectomy with a contralateral Wise-pattern mastopexy with inferior pedicle for symmetry. This technique is ideal for patients with large, ptotic breasts who desire breast conservation with immediate reconstruction.

## METHODS

**Figure 1:**
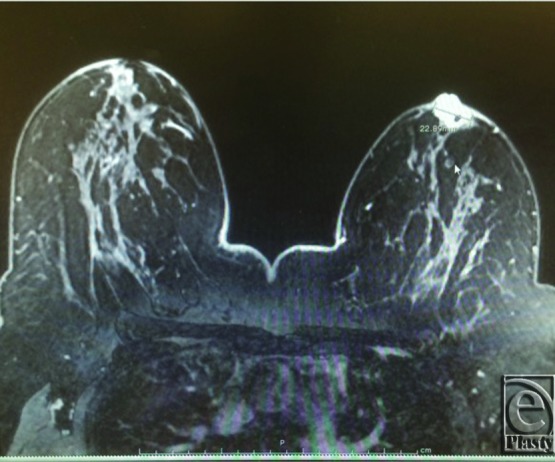
Preoperative MRI.

A 47-year-old woman with size 38 DD breasts presented with a palpable 2-cm subareolar mass of the left breast. It was mammographically occult but immediately deep to NAC on the magnetic resonance image (Fig 1). Ultrasound-guided core biopsy was positive for ER/PR positive, HER2 negative invasive ductal carcinoma. All options for tumor clearance and reconstruction were discussed preoperatively, and the patient decided to undergo breast conservation with contralateral mastopexy for symmetry.

**Figure 2:**
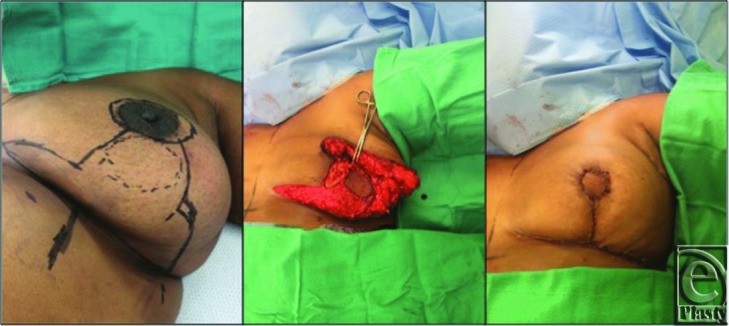
Preoperative markings, Neo-NAC and dermoglandular pedicle, and closure.

We performed preoperative markings for bilateral Wise-pattern mastopexy with inferior pedicle prior to surgical oncology performing left lumpectomy with NAC excision and a sentinel lymph node biopsy. The lumpectomy specimen weight was 108 g. Immediate left breast reconstruction was performed with an inferomedial pedicle island flap (Fig 2). The pedicle was deepithelialized in a reverse L fashion, with the inferior portion of the L extending medially, sparing the neo-NAC skin island. This had a 4-cm diameter and was placed 6 cm above the inframammary fold (IMF). The configuration of the reversed L was necessary to rotate the skin paddle and preserve medial fullness. An additional 30 g of breast tissue was excised laterally for contour and symmetry, the neo-NAC was rotated into the defect, and an inverted-T closure was performed.

The right breast, which had more glandular ptosis, but appeared 50 to 75 g heavier than the left, was approached with a similar Wise-pattern mastopexy with inferior L-shaped pedicle with 6-cm vertical limbs, and plication of the dermal pedicle to 6 cm to match the reconstructed left side. The nipple was raised 2 cm to match the reconstructed left side. A total of 205 g of tissue was removed from the right breast.

## RESULTS

**Figure 3:**
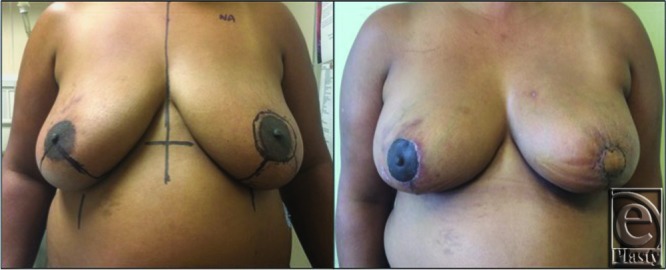
Preoperative and postoperative pictures.

The patient showed excellent symmetry at the conclusion of the procedure. Final pathology demonstrated complete excision of the tumor with negative margins. The entire neo-NAC skin island was viable postoperatively. On follow-up, the surgical site demonstrated good healing with preserved symmetry and viable neo-NAC skin (Fig 3).

## DISCUSSION

The NAC, and its central position on the breast mound, is a defining feature of the breast and must be addressed in reconstruction. This makes immediate breast reconstruction in the setting of central lumpectomy with NAC excision challenging.

Direct closure of the defect results in an abnormal contour with excess skin and bulk medially and laterally. In addition, a purse string closure typically results in a small, open, central skin defect that requires a long duration for healing. These pitfalls make volume displacement and replacement techniques more ideal.

Large-breasted patients are good candidates for volume displacement procedures, such as the mastopexy with neo-NAC. Shifting volume from the lower pole to the upper pole provides the breast with a more youthful appearance and avoids the need for more prolonged, invasive surgery. The size of the defect is less favorable for direct or purse string closure. The neo-NAC is planned to undergo 3-dimensional (3D) tattooing, as desired by the patient.

Small-breasted patients with an IMF to nipple measurement of less than 8 cm are less likely to be good candidates for this type of procedure and may benefit from mastectomy with autogenous or prosthetic volume replacement surgery. The risks of free flap procedures have been well documented, including longer anesthetic time, flap failure, fat necrosis, and the need for multiple revisions. Immediate, single-stage prosthetic reconstruction has also been described, but patients who undergo NAC excision are often not good candidates.

Our approach fulfilled the patient's desire to have a 1-stage procedure. It also demonstrated that immediate reconstruction with contralateral reduction mammoplasty can provide an excellent cosmetic outcome in patients with large, ptotic breasts and central defects following oncologic tumor resection. Other options for autogenous or implant-based reconstruction are still available in the event of recurrence or patient dissatisfaction. Single-procedure advantages include patient convenience, single anesthetic, and the ability to reconstruct a breast that has not yet been affected by radiation.

While this procedure produced an excellent result for the patient, it is not without limitations. Adjuvant radiation therapy can result in retraction and decreased volume of the irradiated breast. The placement of a skin island as a neo-NAC during the procedure could be problematic, and radiation contraction and fibrosis are unpredictable. Offsetting of the neo-NAC skin island would produce a less desirable result. Planned 3D tattooing of the nipple will lack projection but provide a natural appearance.

In summary, we report that immediate reconstruction of an NAC lumpectomy defect with a L-based skin paddle flap and contralateral reduction mammoplasty provides an excellent cosmetic outcome in patients with large, ptotic breasts and central defects following oncologic tumor resection.
